# Evaluation of Veterinary Prescription of Gastroprotectants in Dogs in Spain

**DOI:** 10.3390/vetsci13010061

**Published:** 2026-01-08

**Authors:** Patricia Olmeda, Carmen Rey, Fernando Rodríguez-Franco, Stanley L. Marks, Mercedes García-Sancho, David Díaz-Regañón, Alejandra Villaescusa, Ángel Sainz

**Affiliations:** 1 Department of Animal Medicine and Surgery, College of Veterinary Medicine, Complutense University of Madrid, 28040 Madrid, Spain; patolm01@ucm.es (P.O.); carmenreymateos@gmail.com (C.R.); mercgarc@ucm.es (M.G.-S.); drdiazreganon@ucm.es (D.D.-R.); alejandrav@ucm.es (A.V.); 2 Department of Medicine and Epidemiology, School of Veterinary Medicine, University of California, Davis, CA 95616, USA; slmarks@ucdavis.edu

**Keywords:** proton pump inhibitor, acid suppressants, canine, survey, gastroesophageal reflux, famotidine, omeprazole

## Abstract

The overprescription of gastroprotectants, in particular acid suppressants in dogs, is of increasing concern in veterinary medicine. There have been specific guidelines published to document the appropriate use of this class of drugs; however, the injudicious use of gastroprotectants continues to be a concern and is often not evidence-based. The primary objective of the present study was to evaluate the veterinary prescription of gastroprotectants for dogs in Spain. A survey employing a snowball recruitment effect was distributed among small animal medicine veterinarians practicing in Spain. A total of 265 veterinarians participated in the survey. Proton pump inhibitors (PPIs) were found to be the most commonly prescribed gastroprotectant utilised by 50.6% of the participants. Veterinarians with fewer years of clinical experience and those focusing on the fields of internal medicine, emergency, and anaesthesia were more likely to adhere to evidence-based guidelines in their prescribing practices. Those who prescribed gastroprotectants less frequently tended to rely on PPIs and on international consensus guidelines. Although the main indications in which Spanish veterinarians used gastroprotectants was supported by scientific evidence, the injudicious administration of this class of drugs for disorders lacking robust scientific evidence or recommendations was well documented.

## Simple Summary

Many medications designed to protect the gastrointestinal tract are given to dogs even when they are not really needed. In this study, we asked veterinarians in Spain who work with dogs about how and why they use these medications. A total of 265 professionals completed the questionnaire. Most of them reported that they regularly prescribed drugs that reduce stomach acid. Veterinarians with fewer years of experience, and those working in areas such as internal medicine, emergency or anaesthesia, were more likely to follow scientific guidelines when deciding whether to use these drugs. Those who prescribed them less often tended to rely more on international expert guidelines. Overall, even though many of the reasons veterinarians gave for using gastro-protective medications were supported by scientific evidence, the study also revealed that these drugs are still commonly used in situations where the benefits are uncertain.

## 1. Introduction

The overprescription of gastroprotectants in dogs is of increasing concern in veterinary medicine [1]. These drugs are essential to reduce gastric acid secretion and treat gastrointestinal ulceration and erosion (GUE) and gastroesophageal reflux-associated oesophagitis; however, appropriate clinical doses and length of gastroprotective therapy in dogs has not been fully defined [2,3,4,5,6]. The current scientific evidence lacks sufficient support for their routine use for the prevention of GUE associated with non-steroidal anti-inflammatory drugs (NSAIDs) or glucocorticoid administration [7,8,9,10,11], in chronic kidney disease (CKD) [4,12], pancreatitis [4], and treatment of *Helicobacter pylori* [4] not associated with GUE in dogs.

Although proton pump inhibitors (PPIs) have historically been considered relatively safe, recent studies have raised concerns about their prolonged use in light of their potential adverse effects [4,13,14]. Most of the clinical research has been conducted in healthy research dogs with findings often extrapolated from the human literature, further contributing to suboptimal clinical practice in clinically ill canine patients [6,15].

The overprescription of gastroprotectants in veterinary medicine has been poorly studied and is suspected to be common practice, especially with acid suppressants such as omeprazole and famotidine, which are considered relatively safe and affordable. This can lead to unnecessary costs to owners, adverse clinical signs associated with drug administration, and possible prescribing errors [1,4], similar to that documented in human patients [16,17].

In 2018, the American College of Veterinary Internal Medicine (ACVIM) published a consensus statement questioning the routine use of gastroprotectants in dogs and cats without additional risk factors of GUE or gastrointestinal (GI) bleeding and promoting the judicious administration of gastroprotectants based on scientific evidence [4]. Publication of this consensus statement and clinical audits in veterinary practices have improved the prescribing practices of acid suppressants such as omeprazole [16,18].

The primary objective of the present study was to evaluate the veterinary prescription of gastroprotectants in dogs in Spain. The principal aim was to analyse the frequency of prescription of these drugs by veterinarians, identify the most commonly prescribed gastroprotectants, and investigate potential reasons associated with the inappropriate or excessive use of these medications, including veterinarian experience, clinical field, and type of veterinary practice. We hypothesised that the appropriate and timely prescription of gastroprotectants among Spanish veterinarians has improved over time, but that the injudicious administration of these drugs persists in certain practice settings.

## 2. Materials and Methods

### 2.1. Study Design and Setting

A descriptive cross-sectional study design utilising a survey instrument to evaluate the veterinary prescription of gastroprotectants in dogs in Spain between 31 January and 21 March 2023, was conducted.

### 2.2. Survey

A survey was developed following the existing literature on the prescription of gastroprotectants in veterinary medicine [2,4,6]. The questionnaire comprised 15 multiple choice, single choice, or open-ended format questions and was reviewed for clarity and content validity by five small animal veterinarians prior to distribution, with minor revisions made following their feedback (Appendix A).

The survey was conducted using the Google Forms^®^ (Mountain View, CA, USA) platform which enables the creation of bespoke questionnaires and the generation of automated results. The survey encompassed a range of topics, including demographics, the prevalence of gastroprotectant prescription, the most commonly prescribed gastroprotectants and the rationale behind the selection, the factors impeding the prescription of the preferred gastroprotectant, the frequency of prescribing multiple gastroprotectants concurrently in the same dog, the circumstances under which a gastroprotectant was prescribed, the perceived safety of gastroprotectants (very safe: drug with known pharmacokinetics and no to mild adverse effects; safe: drug with known pharmacokinetics and few manageable adverse effects; unsafe: drug with poorly defined pharmacokinetics and potentially serious adverse effects), and the association of adverse effects following their use (Appendix A). Participants could select all the survey options that applied to their reasons for their selection or for the factors impeding the prescription of the preferred gastroprotectant. The data were subsequently organised and stored in Microsoft Office Excel^®^ (Redmond, WA, USA) for further statistical analysis.

No ethical approval was required for this procedure, neither in the national or EU legal system as enrolment was voluntary and the participants consented to anonymous information according to Regulation (EU) 2016/679 of the European Parliament and of the Council of 27 April 2016, and Organic Law 3/2018, of 5 December 2018, on the Protection of Personal Data and the Guarantee of Digital Rights.

### 2.3. Participants and Sampling

The target population of this survey comprised small animal medicine veterinarians practicing in Spain. A non-probability snowball sampling approach was used to recruit participants. The survey was distributed through social networks (WhatsApp^®^, Menlo Park, CA, USA) and messaging platforms to a randomly selected sample of veterinarians from Spain. The survey was conducted in accordance with the principles of voluntary and anonymous participation. Those veterinarians who participated were informed of the purpose of the study and the confidentiality of the data obtained.

### 2.4. Statistical Methods

To assess the associations between categorical variables, the chi-square test or Fisher’s exact test was applied depending on the conditions. To analyse the relationships between numerical variables and ordinal categorical variables, Spearman’s correlation was employed. Furthermore, when numerical variables were crossed with pure categorical variables, Student’s *t*-test or analysis of variance (ANOVA) was employed. Similarly, when ordinal categorical variables were crossed with one another, Spearman’s correlation was employed to evaluate the relationship between them. In instances where ordinal categorical variables were crossed with categorical variables, the Wilcoxon test or the Kruskal–Wallis test were employed, as appropriate. Subsequently, a multivariate analysis was conducted. Three variables which showed significant differences were selected as independent variables to construct decision trees using an exhaustive CHAID algorithm.

The level of statistical significance was set at *p* < 0.05. Multivariate analyses were conducted using IBM SPSS software, version 29 (IBM Corp, Armonk, NY, USA). The remaining statistical analyses were conducted using SAS software, version 9.4 (SAS Institute, Cary, NC, USA).

## 3. Results

### 3.1. Study Population

The survey was answered by 265 participants with a mean age of 40.5 years (range: 24–63 years). In terms of clinical experience, 22.6% had <5 years of experience, 20.8% had between 5 and 10 years of experience, and 56.6% had >10 years of experience. The most common primary clinical interest (not only restricted to ACVIM/EBVS Diplomates) was small animal internal medicine with 85 participants (32.1%) identified. General practice (*n* = 74; 27.9%), surgery (*n* = 36; 13.6%), emergency medicine (*n* = 21; 7.9%), and anaesthesia (*n* = 14; 5.3%) were the next most common clinical fields of practice. A smaller number of participants had primary clinical interests in oncology (*n* = 9; 3.4%), dermatology (*n* = 6; 2.3%), cardiology (*n* = 5; 1.9%), ophthalmology (*n* = 5; 1.9%), diagnostic imaging (*n* = 4; 1.5%), neurology (*n* = 3; 1.1%), reproduction (*n* = 2; 0.8%) and dentistry (*n* = 1; 0.4%).

### 3.2. Gastroprotectant Prescription

Overall, 35.5% of the veterinarians surveyed (*n* = 94) prescribed gastroprotectants in <10% of the cases, 37.4% (*n* = 99) in a range of 10–30% of cases, 21.5% (*n* = 57) in a range of 30–50% of cases, and 5.7% (*n* = 15) in >50% of cases. Proton pump inhibitors represented the most common type of gastroprotectant chosen by 50.6% of the participants (*n* = 134) followed by antacids by 28.3% of participants (*n* = 75), histamine-2 Receptor Antagonists (H2RAs) chosen by 18.5% of the participants (*n* = 49), and sucralfate chosen by 2.6% of the participants (*n* = 7). None of the participants reported using misoprostol (*n* = 0). A total of 35.9% of the participants (*n* = 95) indicated that they made their selection on the basis of scientific evidence, 33.2% (*n* = 88) on the superior efficacy of their preferred choice in comparison to other available options, 29.8% (*n* = 79) on the availability of a diverse range of commercial formulations, 20.8% (*n* = 55) on the drug’s lower cost in comparison to other available options, 19.6% (*n* = 52) on a perceived lower incidence of adverse effects, and 2.3% (*n* = 6) on the ease of administration.

Omeprazole was the most frequently prescribed acid suppressant (*n* = 184; 69.4%), followed by famotidine (*n* = 79; 29.8%), esomeprazole (*n* = 1; 0.4%) and pantoprazole (*n* = 1; 0.4%). Lastly, 82 participants (30.9%) utilised combinations of gastroprotectants, in the same dog, of which 39.0% (*n* = 32) combined omeprazole with an antacid, 35.4% (*n* = 29) combined omeprazole with sucralfate, 26.8% (*n* = 22) combined famotidine with an antacid, 8.5% (*n* = 7) combined famotidine with sucralfate, 4.9% (*n* = 4) combined famotidine with omeprazole, and 2.4% (*n* = 2) combined pantoprazole with an antacid.

#### 3.2.1. Prescription of Gastroprotectants in Different Clinical Settings

A total of 92.8% of respondents indicated that they used these gastroprotectants for the treatment of GUE (*n* = 246), 83.8% for the treatment of reflux oesophagitis (*n* = 222), 49.8% for the management of non-erosive gastritis (*n* = 132), 44.2% for prevention of GUE in association with NSAID administration (*n* = 117), 43.4% for dogs with CKD (*n* = 115), 38.9% for dogs with *Helicobacter* spp. infection (*n* = 103), 31.3% for dogs with pancreatitis and without GUE (*n* = 83), 29.8% for management of thrombocytopenia-induced gastrointestinal haemorrhage (*n* = 79), 28.7% for prevention of GUE in association with glucocorticoid administration (*n* = 76), 20.4% for canine patients in the intensive care unit (ICU) (*n* = 54), 11.3% for prevention of anaesthesia-associated reflux oesophagitis (*n* = 30), 4.9% for empiric management of stressed dogs (*n* = 13), and 4.5% for dogs with hepatic disease without GUE or apparent risk of GI bleeding (*n* = 12).

#### 3.2.2. Perception of Safety and Adverse Effects of Gastroprotectants

A total of 183 respondents (69.1%) rated these drugs as safe, although the necessity for further studies to precisely define their efficacy was emphasised. Furthermore, 30.6% (*n* = 81) of respondents rated them as very safe, and a sole veterinarian (0.4%) deemed these drugs to be unsafe. Adverse effects following the use of gastroprotectant therapy were observed by 12.8% of the respondents (*n* = 34). The adverse effects included diarrhoea (*n* = 12; 35.3%), vomiting (*n* = 5; 14.7%), allergic reaction (*n* = 4; 11.8%), intestinal dysbiosis (*n* = 3; 8.8%), rebound gastric acid hypersecretion (*n* = 3; 8.8%), gastric mucosal hyperplasia (*n* = 2; 5.9%), and vertigo (*n* = 1; 3%).

#### 3.2.3. Training on the Use of Gastroprotectants

A need for further training on the latest trends in the use of gastroprotectants was indicated by 94.3% of participants (*n* = 250).

### 3.3. Associations Between Different Variables and the Prescription of Gastroprotectants

Due to the high correlation between the age of the participants and the length of clinical experience (rs = 0.86330; *p* < 0.0001), the analysis of the statistically significant associations with the remaining questions of the survey were completed using the latter variable (Table 1). The potential association between the clinical field of practice and gastroprotectant prescribing patterns was also evaluated, with the statistically significant results presented in Table 2. The statistical significance of the associations with the other variables was also determined based on the proportion of cases in which clinicians prescribed gastroprotectants in either <10% or >50% of cases (Table 3). Depending on the gastroprotective agent of preference, significant associations were identified with other prescription variables in the survey (Table 4).

### 3.4. Multivariate Analyses

**Figure 1:**
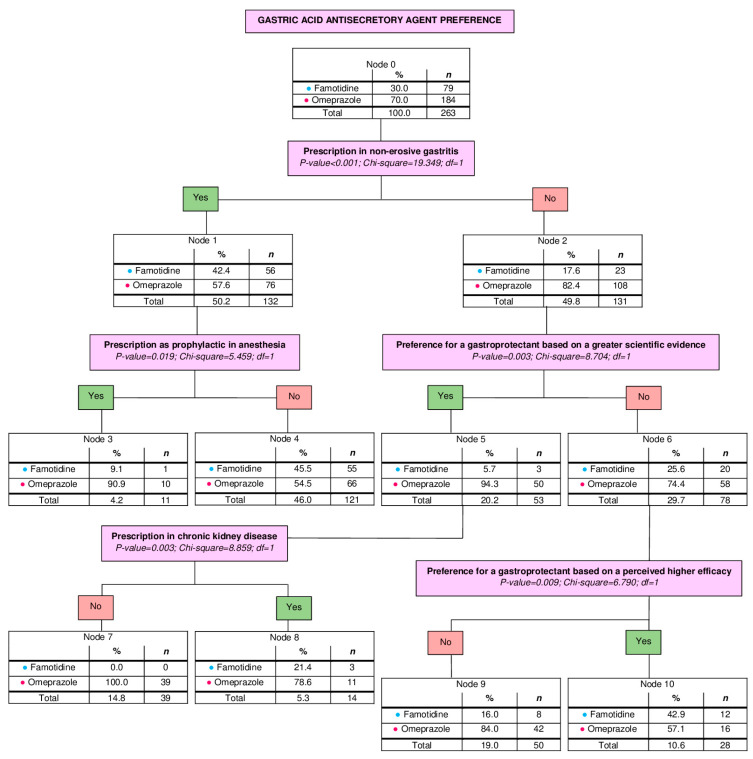
Decision tree based on the gastric acid antisecretory preference between omeprazole and famotidine. Estimated risk: 0.300; Standard error: 0.028. *n*, number of veterinarians.

Multivariate analyses were performed using decision trees, three of which were statistically significant. One of them evaluated the preference of acid suppressant therapy between omeprazole (70%, *n* = 184) and famotidine (30%, *n* = 79). The number of nodes obtained was 11 (from 0 to 10). The number of terminal nodes was 6. The depth was 3 levels. Significant results derived from this first tree are shown in Figure 1.

**Figure 2:**
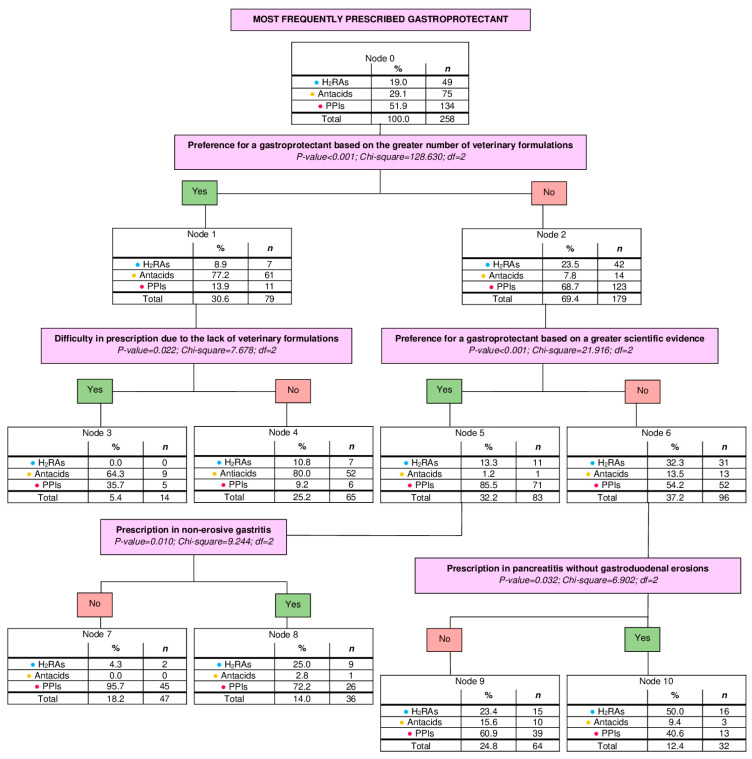
Decision tree based on the preferences for specific categories of gastroprotectants, between H2RAs, antiacids and PPIs. Estimated risk: 0.275; Standard error: 0.028. *n*, number of veterinarians; H2RAs, histamine type-2 receptor antagonists; PPIs, proton pump inhibitors.

The second analysis evaluated the preferences for specific categories of gastroprotectants, including H2RAs (19%, *n* = 49), antacids (29.1%, *n* = 75) and PPIs (51.9%, *n* = 134). Preference for sucralfate was excluded from this analysis in light of its low representation (2.6%, *n* = 7). The number of nodes obtained was 11 (from 0 to 10). The number of terminal nodes was 6. The depth was 3 levels. Significant results derived from the second tree are shown in Figure 2.

**Figure 3:**
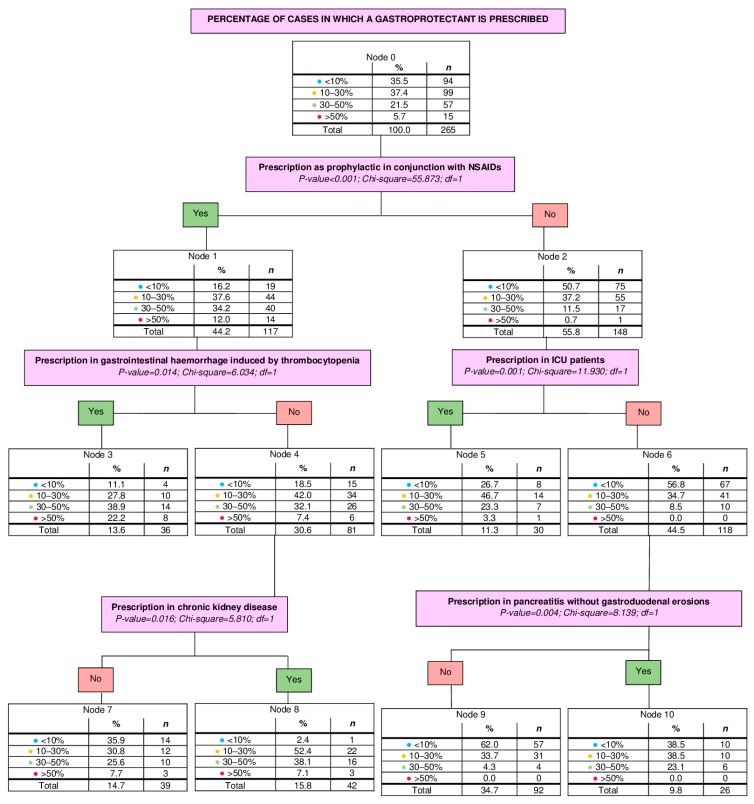
Decision tree based on the percentage of cases in which a gastroprotectant is prescribed. Estimated risk: 0.506; Standard error: 0.031. *n*, number of veterinarians, NSAIDs, non-steroidal anti-inflammatory drugs; ICU, intensive care unit; <10%, percentage of prescription of gastroprotectants in less than 10% of cases; 10–30%, percentage of prescription of gastroprotectants between 10 and 30% of cases; 30–50%, percentage of prescription of gastroprotectants between 30 and 50% of cases; >50%, percentage of prescription of gastroprotectants in more than 50% of cases.

The third analysis evaluated the percentage of cases in which a gastroprotectant was prescribed. Overall, 35.5% (*n* = 94) of participants prescribed gastroprotectants in <10% of cases, 37.4% (*n* = 99) between 10 and 30%, 21.5% (*n* = 57) between 30 and 50% and 5.7% (*n* = 15) in >50% of cases. The number of nodes obtained was 11 (from 0 to 10). The number of terminal nodes was 6. The depth was 3 levels. Significant results derived from the third tree are shown in Figure 3.

## 4. Discussion

This study along with a previously published study [18] represent the first comprehensive evaluations of the veterinary prescription of gastroprotectants in dogs in Spain. The results demonstrated a diversity in the prescription of gastroprotectants in clinical practice among the surveyed veterinarians even though most respondents indicated a preference for more limited use of these drugs.

Most respondents recommended omeprazole as a gastroprotectant compared to famotidine in line with scientific evidence, similar to the findings of previous studies [2]. The selection of less effective antacids by some veterinarians may be attributed to the ease of their accessibility, status as the oldest pharmaceuticals on the market, and their lower cost. In contrast, the minority of participants who prescribed sucralfate more frequently may have prescribed it for its cytoprotective effects [4]. The absence of misoprostol use among respondents may reflect its potential side effects and significant safety concerns associated with its use, particularly its abortifacient effects in people, and more restricted indications compared to other gastroprotectants commonly used in canine practice.

PPIs have been shown to be more effective in raising intragastric pH compared to H2RAs and are the preferred choice when dealing with GUE and reflux oesophagitis [4,19,20,21]. The percentage of veterinarians combining gastroprotectants was around 30%, in agreement with former studies [1,2]. There is a perception that implementing H2RAs with PPIs will result in a greater onset of acid suppression; however, there is well documented evidence that the co-administration of H2RAs with PPIs decreases the acid suppressant effectiveness of PPIs [2,4,22]. Omeprazole was by far the most common PPI used compared to esomeprazole and pantoprazole. This finding was attributed to the fact that omeprazole has broader availability and lower cost compared to other acid suppressants in Spain [23].

The injudicious use of gastroprotectants was well documented in a Portuguese study [2] that showed the improper use of gastroprotectants to prevent corticosteroid-associated GUE, to manage dogs with pancreatitis without GUE, for management of dogs with CKD, for dogs with GI bleeding induced by thrombocytopenia, for dogs with liver disease without risk of GI bleeding, and in non-erosive gastritis. Another controversial indication for which gastroprotectants are frequently prescribed and was assessed in our study pertains to the administration of PPIs for the prevention of NSAIDs-associated GUE [7,9,10,24]. Our study showed that fewer veterinarians prescribed gastroprotectants injudiciously, in particular for the management of non-erosive gastritis, compared with the findings reported in the previously mentioned study [2] (49.8% vs. 89.5%); however, this percentage is still too high. It is plausible that the observed decline in prescription rates between the two studies reflects an increasing awareness of the judicious use of gastroprotectants following the publication of the ACVIM consensus statement [4]. The initial study was conducted in 2020 where only 43.5% of Portuguese veterinarians who completed the survey were aware of the ACVIM consensus statement, while the present study was conducted in 2023. A previous investigation conducted in a veterinary teaching hospital in Spain reported that 84.2% of the veterinarians surveyed were aware of the ACVIM consensus statement [18]; however, this data may not be fully representative of the broader clinician population because the study in question was conducted at a single veterinary teaching hospital in Madrid.

Some of the adverse effects detected by participants in our study have previously been associated with the administration of gastroprotectants, in particular PPIs. Although the acid suppressants are considered relatively safe drugs, adverse effects such as alteration of the GI microbiome in dogs [7,17], vomiting, diarrhoea, rebound gastric acid hypersecretion, hypergastrinemia and development of hyperplastic gastritis have been previously reported [2,13,14,25]. The proportion of veterinarians reporting adverse reactions in the present study may be partially explained by the widespread and, in some cases, prolonged or prophylactic use of gastroprotectants in clinical scenarios not supported by strong evidence. Inappropriate dosing, extended treatment duration, or the use of combination therapy may further increase the risk of adverse effects. It should also be acknowledged that some of the reported events may not represent true adverse reactions to the drug, but rather clinical signs attributable to the underlying disease being treated.

It is notable that despite most veterinarians being aware of the ACVIM consensus statement on the use of gastroprotectants in small animals, 94.3% indicated a need for further training in this area. This may be attributed to the evolving nature of veterinary medicine and the conviction that ongoing training is essential to remain abreast of the latest clinical standards, as confirmed in several studies [16,18,26]. Veterinarians with more years of clinical experience were found to prescribe famotidine more frequently compared to those with less experience. Previous studies have suggested that this group of clinicians may be less current on the scientific literature or more likely to base their decisions on their clinical experience [2,26]. In contrast, younger veterinarians tend to be more familiar with recent scientific evidence and, particularly in the early stages of their careers, may be more inclined to actively seek updated information, clinical guidelines, and consensus statements when prescribing medications, potentially due to their lack of experience. Additionally, the undergraduate and postgraduate training received by younger veterinarians is likely to be more closely aligned with current evidence-based recommendations than that received by their more experienced counterparts. The increased use of evidence-based resources, coupled with a greater openness to ongoing education and training, may be contributing to the more guideline-adherent prescribing patterns observed among less experienced veterinarians [26,27].

Veterinarians in the fields of anaesthesia and in internal medicine tended to prescribe PPIs more frequently, basing their prescribing criteria on scientific evidence to a greater extent. In addition, this group of veterinarians were also more likely to prescribe gastroprotectants in appropriate clinical scenarios compared to colleagues working in other areas of clinical practice. Compared to their counterparts, veterinarians working in ICU settings preferred omeprazole as an acid suppressant. This may be attributed to the fact that this clinical field is represented by younger veterinarians that are likely more familiar with the latest scientific trends. It is noteworthy that a considerable number of studies assessing the efficacy of acid suppressants for prevention of reflux oesophagitis in dogs under general anaesthesia have been conducted with PPIs, which could have played a significant role in influencing the prescription of these drugs by anaesthetists [28,29,30,31]. In contrast, veterinarians working in general practice were less likely to prescribe omeprazole and more likely to prescribe famotidine, and were also more prone to prescribing these drugs in situations lacking scientific support [4].

A higher percentage of veterinarians working in anaesthesia and internal medicine reported adverse effects, which may be due to the types of cases they manage and their individual clinical interests, particularly regarding gastrointestinal medicine. This may be attributed to their greater familiarity with these drugs and consequently their adverse effects. Likewise, those working in anaesthesia are more familiar with the side effects of acid suppressants because of the inherent risk of reflux oesophagitis associated with anaesthetic procedures [4,29,30].

It should be noted that the frequency of gastroprotectant use reflected general prescribing patterns that may have been influenced by the caseload and types of cases managed by the participants. However, no statistically significant associations were identified between gastroprotectant prescribing frequency and the veterinarian’s self-reported area of interest. Nonetheless, these contextual factors should be considered when interpreting prescribing frequency data, as they may partly explain the variability observed among respondents. Veterinarians who prescribed gastroprotectants in lower proportions showed better selection and knowledge in their choice of judicious administration of gastroprotectants supported by scientific evidence [4,6].

A significant positive correlation was identified between participants who prescribed antacids with greater frequency and those who prescribed gastroprotectants in combination with NSAIDs for prevention of GUE despite the lack of evidence to support the concurrent use of gastroprotectants and NSAIDs in clinical practice [4,7,9,10,24,32]. Additionally, a positive correlation was identified between the preference for H2RAs over PPIs and the utilisation of gastroprotectants in clinical contexts that lacked scientific evidence to support their use. As previously discussed, those veterinarians who prescribed antacids or H2RAs may be unaware of the most recent scientific advances in this area. This observation is supported by the finding that all the veterinarians who prescribed these two types of drugs most frequently indicated a need for additional training, suggesting an awareness of their lack of knowledge in this area. The multivariate analyses conducted to circumvent potential confounding factors corroborated some of the aforementioned results. Clinicians who preferentially prescribed omeprazole did so primarily on the basis of its greater scientific basis and in more appropriate clinical situations. However, the prescription of PPIs also poses greater prescribing challenges due to the lack of veterinary formulations in Spain. Furthermore, veterinarians who prescribed gastroprotectants less frequently did so in a more judicious fashion.

It is crucial to consider the limitations of this study when interpreting the results. As a non-probability snowball sampling strategy was used and the questionnaire was distributed primarily via social media and professional messaging platforms, there is an inherent risk of selection bias. It is acknowledged that veterinarians who choose to participate may not be a fully representative sample of the wider veterinary population in Spain. In order to reduce this risk, the survey was initially disseminated across a range of professional networks, including veterinarians from different geographic regions, age groups, and types of veterinary practice, and remained open for a defined period to maximise participation. However, the generalisability of the findings may be limited and should be interpreted with caution. In addition, although the questionnaire was reviewed by five small animal veterinarians to ensure clarity and content relevance, it did not undergo formal validation.

Furthermore, the act of requesting that veterinarians indicate their gastroprotectant prescriptions in various clinical scenarios may have influenced their responses and may have resulted in an observer response effect, where respondents may have adjusted their responses to align with their perceived expectations or desired outcomes.

## 5. Conclusions

Proton pump inhibitors, particularly omeprazole, are the most commonly prescribed gastroprotectants in dogs by small animal veterinarians in Spain. However, limited availability of these drugs restricts the range of treatment options. It was observed that veterinarians with fewer years of clinical experience and those focusing on internal medicine, emergency, and anaesthesia were more likely to adhere to evidence-based guidelines in their prescribing practices. In addition, those veterinarians who prescribed gastroprotectants less frequently tended to rely on PPIs and on published guidelines.

The principal indication for the use of gastroprotectants among small animal veterinarians in Spain is the treatment of GUE and reflux oesophagitis, a position supported by scientific evidence. However, these medications are also frequently inappropriately prescribed for disorders such as non-erosive gastritis and prophylaxis of NSAID-associated GUE, despite a lack of robust evidence. Ongoing education among veterinarians is recommended to ensure they remain informed of the latest research and trends in the use of gastroprotectants.

## Figures and Tables

**Table 1:** Statistically significant associations according to the length of clinical experience of the participants.

	<5 Years (%)	5–10 Years (%)	>10 Years (%)	*p*-Value
Clinical field of practice				
Anaesthesia	11.7	7.3	2	0.004
General practice	6.7	16.4	40.7	<0.001
ICU	13.3	18.2	2	<0.001
Acid Suppressant Preference				0.003
Famotidine	16.7	25.5	37.2	
Omeprazole	83.3	74.5	62.8	
Clinical Setting				
Prophylaxis in conjunction with glucocorticoids	18.3	20	36	0.004
Prophylaxis in conjunction with NSAIDs	35	36.4	50.7	0.018
CKD	35	38.2	48.7	0.048
Thrombocytopenia-induced GI bleeding	36.7	36.4	24.7	0.045

CKD, chronic kidney disease; GI, gastrointestinal; ICU, intensive care unit; NSAIDs, non-steroidal anti-inflammatory drugs.

**Table 2:** Statistically significant associations between clinical field of practice and the prescription of gastroprotectants.

	% of the Corresponding Clinical Field	% of Veterinarians from Other Clinical Field	*p*-Value
Gastroprotectant Selections			
Anaesthesia			0.001
PPIs	92.7	48.2	
Sucralfate	7.1	2.4	
General practice			<0.0001
Antacids	48.7	20.4	
PPIs	24.3	60.7	
Internal medicine			0.036
PPIs	62.4	45	
Antacids	18.8	32.8	
Prescription reason			
Anaesthesia			
Greater scientific evidence	71.4	33.9	0.004
General practice			
Fewer adverse effects	28.4	16.2	0.026
Increased availability	43.2	24.6	0.003
Greater scientific evidence	14.9	44	<0.0001
Internal medicine			
Greater scientific evidence	51.8	28.3	0.0002
Prescription difficulties			
ICU			
Lack of availability in veterinary medicine	47.6	24.2	0.019
Acid Suppressant selection			
General practice			0.009
Famotidine	41.9	25.4	
Omeprazole	58.1	74.6	
ICU			0.002
Omeprazole	100	32.6	
Gastroprotectant combination prescription			
ICU	52.4	29.1	0.027
Clinical scenarios			
Prophylaxis in anaesthesia			
Anaesthesia	50	9.2	0.0002
ICU	9.5	33.2	0.025
Prophylaxis with glucocorticoids			
General practice	44.6	22.5	0.0004
ICU	4.8	30.7	0.012
Prophylaxis with NSAIDs			
General practice	59.5	38.2	0.002
ICU	4.8	47.5	0.0002
CKD			
General practice	56.8	38.2	0.006
GUE treatment			
Internal medicine	97.7	90.6	0.037
Non-erosive gastritis			
ICU	23.8	52.1	0.013
Pancreatitis without GUE			
ICU	9.5	33.2	0.025
Adverse effects detection			
Anaesthesia	35.7	11.6	0.022
Internal medicine	20	9.4	0.017
Need for training			
Internal medicine	89.4	96.7	0.023

CKD, chronic kidney disease; GUE, gastrointestinal ulceration and erosion; ICU, intensive care unit; NSAIDs, non-steroidal anti-inflammatory drugs; PPIs, proton pump inhibitors.

**Table 3:** Statistically significant associations between prescription frequency and the prescription of gastroprotectants.

	% Prescriptions in <10% of Cases	% Prescriptions in >50% of Cases	*p*-Value
Gastroprotectant preferences			0.002
PPIs	62.8	33.3	
Antacids	23.4	53.3	
Prescription reason			0.017
Greater scientific evidence	45.7	20	
Antisecretory agent selection			0.001
Omeprazole	82.6	73.3	
Famotidine	17.4	26.7	
Clinical scenarios			
Prophylaxis with glucocorticoids	16	66.7	<0.0001
Prophylaxis with NSAIDs	20.2	93.3	<0.0001
Stressed animals	2.1	26.7	0.028
Non-erosive gastritis	35.1	53.3	0.001
CKD	24.5	53.3	<0.0001
Pancreatitis without GUE	14.9	46.7	<0.0001
Thrombocytopenia-induced GI haemorrhage	21.3	53.3	0.014
*Helicobacter* spp. Infection	28.7	60	0.006
ICU animals	10.6	33.3	0.004
Need for training	90.4	100	0.01

CKD, chronic kidney disease; GI, gastrointestinal; GUE, gastrointestinal ulceration or erosion; ICU, intensive care unit; NSAIDs, non-steroidal anti-inflammatory drugs; PPIs, proton pump inhibitors.

**Table 4:** Statistically significant associations between gastroprotectant preferences and the prescription of gastroprotectants.

	H2RAs (%)	Antacids (%)	PPIs (%)	*p*-Value
Prescription reason				
Higher efficacy	46.9	18.7	35.8	0.003
More economical	30.6	9.3	24.6	0.007
Fewer adverse effects	22.5	33.3	11.2	0.001
Increased availability	14.3	81.3	8.2	<0.0001
Greater scientific evidence	24.5	2.7	56	<0.0001
Difficulty of prescription				
Not available in veterinary market	18.4	14.7	35.1	0.002
Clinical scenarios				
Prophylaxis with NSAIDs	53.1	57.3	34.3	0.002
Prophylaxis with glucocorticoids	40.8	36	20.9	0.009
Non-erosive gastritis	69.4	48	44.1	0.009
CKD	59.2	44	38.1	0.039
Pancreatitis without GUE	51	36	23.1	0.001
*Helicobacter* spp. infection	55.1	36	33.6	0.026
Adverse effects detection	6.1	4	20.2	0.001
Need for training	100	100	88.8	0.0003

CKD, chronic kidney disease; H2RAs, histamine type-2 receptor antagonists; GUE, gastrointestinal ulceration or erosion; NSAIDs, non-steroidal anti-inflammatory drugs; PPIs, proton pump inhibitors.

## Data Availability

The original contributions presented in this study are included in the article. Further inquiries can be directed to the corresponding authors.

## Abbreviations

The following abbreviations are used in this manuscript.

ACVIM	American College of Veterinary Internal Medicine
CKD	chronic kidney disease
GI	Gastrointestinal
GUE	gastroduodenal ulceration and erosion
H2RAs	histamine-2 Receptor Antagonists
ICU	intensive care unit
NSAIDs	non-steroidal anti-inflammatory drugs
PPIs	proton pump inhibitors

## Appendix A

Survey employed to evaluate the veterinary prescription of gastroprotectants in dogs in Spain.

1. Age of participant (years)
2. How many years have you been working in a small animal medicine?
  - Less than 5 years
  - Between 5 and 10 years
  - More than 10 years
3. What is your main area of interest?
  - General Practice
  - Internal Medicine
  - Surgery
  - Oncology
  - Emergency Medicine
  - Anaesthesia
  - Dentistry
  - Neurology
  - Ophthalmology
  - Dermatology
  - Other:
4. In what percentage of your cases do you prescribe a gastroprotectant in dogs?
  - Less than 10%
  - 10–30%
  - 30–50%
  - More than 50%
5. What gastroprotectants do you most frequently prescribe?
  - Antacids (Drugs containing aluminium hydroxide, magnesium hydroxide, and calcium carbonate including Gastrovet, Vet Gastril, Adiva gastric, etc.).
  - Sucralfate
  - Misoprostol
  - Proton pump inhibitors (omeprazole, pantropazole, esomeprazole, lansoprazole)
  - H2RAs (famotidine) *
6. With respect to the previous question, why? You can answer more than one:
  - They are more effective than the rest
  - They are cheaper
  - They have fewer adverse effects
  - They have more commercial formulations for veterinary use.
  - They have more scientific evidence
  - Other: Please mention your reason
7. Are there any problems that make it difficult to prescribe your preferred gastroprotectant? You can answer more than one:
  - No problem
  - It is not marketed in Veterinary Medicine, only in Human Medicine, and I prefer to prescribe Veterinary gastroprotectants
  - It is difficult to dose
  - Other: Please mention your reason
8. Which gastric acid suppressant do you most frequently prescribe?
  - Famotidine
  - Omeprazole
  - Pantoprazole
  - Esomeprazole
  - Lansoprazole
9. Do you sometimes prescribe more than one gastroprotectant in the same dog?
  - Yes
  - No
10. With respect to the previous question, if you have ticked ‘Yes’, please indicate which ones:
11. In which situations would you prescribe a gastroprotectant? You can answer more than one:
  - Treatment of gastroduodenal ulcer or erosion.
  - Treatment of reflux oesophagitis
  - Prophylactic in conjunction with glucocorticoids
  - Prophylactic in conjunction with NSAIDs
  - In patients with liver disease with no risk of gastrointestinal bleeding
  - In patients with stress
  - In non-erosive gastritis
  - In patients with chronic kidney disease (without evidence of GUE)?
  - Prophylactic in anaesthesia
  - In pancreatitis without gastroduodenal erosions
  - In gastrointestinal haemorrhage induced by thrombocytopenia
  - In patients with *Helicobacter* spp. infection
  - In ICU patients
12. How safe do you think gastroprotectants are?
  - Very safe
  - Safe, although more studies are needed to define efficacy
  - Not very safe
13. Have you associated any adverse effects with the use of gastroprotectants?
  - Yes
  - No
14. With respect to the previous question, if you ticked ‘Yes’, please indicate which ones:
15. Finally, a question about training on gastroprotectants:
  - I think that training on the latest trends in the use of gastroprotectants is necessary
  - I am sufficiently up-to-date on this subject

* Cimetidine and ranitidine are not currently marketed in Spain

## References

[1] Duxbury S., Sorah E., Tolbert M.K. (2022). Evaluation of Proton Pump Inhibitor Administration in Hospitalized Dogs in a Tertiary Referral Hospital. J. Vet. Intern. Med..

[2] Baptista R., Englar R., São Braz B., Leal R.O. (2021). Survey-Based Analysis of Current Trends for Prescribing Gastrointestinal Protectants among Small-Animal General Practitioners in Portugal. Vet. Sci..

[3] Jenkins C.C., DeNovo R.C., Patton C.S., Bright R.M., Rohrbach B.W. (1991). Comparison of Effects of Cimetidine and Omeprazole on Mechanically Created Gastric Ulceration and on Aspirin-Induced Gastritis in Dogs. Am. J. Vet. Res..

[4] Marks S.L., Kook P.H., Papich M.G., Tolbert M.K., Willard M.D. (2018). ACVIM Consensus Statement: Support for Rational Administration of Gastrointestinal Protectants to Dogs and Cats. J. Vet. Intern. Med..

[5] Ostronic A., Gremillion C., Zhang S., Steiner J.M., Tolbert M.K., Gould E.N. (2024). Pharmacodynamics of 2 Dosages of Orally Administered Esomeprazole in Client-owned, Healthy Dogs: A Prospective, Crossover Study. J. Vet. Intern. Med..

[6] Tolbert M.K. (2021). Gastroprotective Therapy. Vet. Clin. N. Am. Small Anim. Pract..

[7] Jones S.M., Gaier A., Enomoto H., Ishii P., Pilla R., Price J., Suchodolski J., Steiner J.M., Papich M.G., Messenger K. (2020). The Effect of Combined Carprofen and Omeprazole Administration on Gastrointestinal Permeability and Inflammation in Dogs. J. Vet. Intern. Med..

[8] Rak M.B., Moyers T.D., Price J.M., Whittemore J.C. (2023). Clinicopathologic and Gastrointestinal Effects of Administration of Prednisone, Prednisone with Omeprazole, or Prednisone with Probiotics to Dogs: A Double-Blind Randomized Trial. J. Vet. Intern. Med..

[9] Shaevitz M.H., Moore G.E., Fulkerson C.M. (2021). A Prospective, Randomized, Placebo-Controlled, Double-Blinded Clinical Trial Comparing the Incidence and Severity of Gastrointestinal Adverse Events in Dogs with Cancer Treated with Piroxicam Alone or in Combination with Omeprazole or Famotidine. J. Am. Vet. Med. Assoc..

[10] Wallace J.L., Syer S., Denou E., de Palma G., Vong L., McKnight W., Jury J., Bolla M., Bercik P., Collins S.M. (2011). Proton Pump Inhibitors Exacerbate NSAID-Induced Small Intestinal Injury by Inducing Dysbiosis. Gastroenterology.

[11] Whittemore J.C., Mooney A.P., Price J.M., Thomason J. (2019). Clinical, Clinicopathologic, and Gastrointestinal Changes from Aspirin, Prednisone, or Combination Treatment in Healthy Research Dogs: A Double-Blind Randomized Trial. J. Vet. Intern. Med..

[12] IRIS Treatment Recommendations for CKD in Dogs. https://www.iris-kidney.com/iris-guidelines-1.

[13] Gil-Vicente L., Martín G., Soler C., Vila A., Saiz M.R., Navarro P.F. (2024). Prospective Randomized Controlled Clinical Trial of the Long-Term Effects of Omeprazole on Healthy Dogs. Animals.

[14] Bazelle J., Threlfall A., Whitley N. (2018). Gastroprotectants in Small Animal Veterinary Practice—A Review of the Evidence. Part 1: Cyto-Protective Drugs. J. Small Anim. Pract..

[15] Gaier A., Price J., Grubb L., Fitzgerald S., Tolbert M.K. (2021). A Prospective, Randomized, Masked, Placebo-Controlled Crossover Study for the Effect of 10 Mg Omeprazole Capsules on Gastric pH in Healthy Dogs. J. Vet. Intern. Med..

[16] Bodnarova T., Hall E., Duplan F. (2022). Prescribing Habits for the Use of Omeprazole as a Gastroprotectant in Dogs in a Veterinary Teaching Hospital. J. Small Anim. Pract..

[17] McCormack R., Olley L., Glanemann B., Swann J.W. (2020). Prospective Observational Study of the Use of Omeprazole and Maropitant Citrate in Veterinary Specialist Care. Sci. Rep..

[18] Sainz Á., García-Sancho M., Villaescusa A., Rodríguez-Franco F., Díaz-Regañón D., Olmeda P., Marks S.L. (2024). Prevalence and Appropriateness of Omeprazole Prescription in Dogs at a Veterinary Teaching Hospital before and after the Publication of the ACVIM Consensus Statement on the Rational Administration of Gastrointestinal Protectants. Front. Vet. Sci..

[19] Bersenas A.M.E., Mathews K.A., Allen D.G., Conlon P.D. (2005). Effects of Ranitidine, Famotidine, Pantoprazole, and Omeprazole on Intragastric pH in Dogs. Am. J. Vet. Res..

[20] Tolbert K., Bissett S., King A., Davidson G., Papich M., Peters E., Degernes L. (2011). Efficacy of Oral Famotidine and 2 Omeprazole Formulations for the Control of Intragastric pH in Dogs. J. Vet. Intern. Med..

[21] Williamson K.K., Willard M.D., Payton M.E., Davis M.S. (2010). Efficacy of Omeprazole versus High-Dose Famotidine for Prevention of Exercise-Induced Gastritis in Racing Alaskan Sled Dogs. J. Vet. Intern. Med..

[22] De Graef J., Woussen-Colle M.C. (1986). Influence of the Stimulation State of the Parietal Cells on the Inhibitory Effect of Omeprazole on Gastric Acid Secretion in Dogs. Gastroenterology.

[23] Saiz Ladera G.M., Pejenaute Labari M.E., García Pascual J.N. (2021). Updating in prescription of proton pump inhibitors. What to do and what not to do. Semergen.

[24] Mabry K., Hill T., Tolbert M.K. (2021). Prevalence of Gastrointestinal Lesions in Dogs Chronically Treated with Nonsteroidal Anti-Inflammatory Drugs. J. Vet. Intern. Med..

[25] Lee H., Kim S., Lee D., Chae Y., Yun T., Yang M.-P., Kang B.-T., Kim S., Kim H. (2023). Case Report: Fundic Gland Polyps Caused by Long-Term Omeprazole Use in a Maltese Dog. Front. Vet. Sci..

[26] Dale V.H.M., Pierce S.E., May S.A. (2013). Motivating Factors and Perceived Barriers to Participating in Continuing Professional Development: A National Survey of Veterinary Surgeons. Vet. Rec..

[27] Springer S., Sandøe P., Grimm H., Corr S.A., Kristensen A.T., Lund T.B. (2021). Managing Conflicting Ethical Concerns in Modern Small Animal Practice-A Comparative Study of Veterinarian’s Decision Ethics in Austria, Denmark and the UK. PLoS ONE.

[28] Lotti F., Twedt D., Warrit K., Bryan S., Vaca C., Krause L., Fukushima K., Boscan P. (2021). Effect of Two Different Pre-Anaesthetic Omeprazole Protocols on Gastroesophageal Reflux Incidence and pH in Dogs. J. Small Anim. Pract..

[29] Mehra J.M., Tolbert M.K., Guadiano P., Steiner J.M., Moore G.E., Lewis M.J. (2023). Double-Blinded Placebo-Controlled Clinical Trial of Prophylactic Omeprazole in Dogs Treated Surgically for Acute Thoracolumbar Intervertebral Disc Extrusion. J. Vet. Intern. Med..

[30] Panti A., Bennett R.C., Corletto F., Brearley J., Jeffery N., Mellanby R.J. (2009). The Effect of Omeprazole on Oesophageal pH in Dogs during Anaesthesia. J. Small Anim. Pract..

[31] Zacuto A.C., Marks S.L., Osborn J., Douthitt K.L., Hollingshead K.L., Hayashi K., Kapatkin A.S., Pypendop B.H., Belafsky P.C. (2012). The Influence of Esomeprazole and Cisapride on Gastroesophageal Reflux during Anesthesia in Dogs. J. Vet. Intern. Med..

[32] Eichstadt L.R., Moore G.E., Childress M.O. (2017). Risk Factors for Treatment-Related Adverse Events in Cancer-Bearing Dogs Receiving Piroxicam. Vet. Comp. Oncol..

